# Ensuring Children and Adolescents Are Not Left Behind

**DOI:** 10.1097/QAI.0000000000001751

**Published:** 2018-07-11

**Authors:** Linda-Gail Bekker, George K. Siberry, Gottfried Hirnschall

**Affiliations:** * The Desmond Tutu HIV Center, University of Cape Town, Cape Town, South Africa;; † U.S. Global AIDS Coordinator (S/GAC), U.S. Department of State, Washington, DC; and; ‡ Department of HIV and Global Hepatitis Programme, World Health Organization, Geneva, Switzerland.

**Keywords:** pediatric HIV, adolescent HIV, HIV research agenda

I hate having AIDS because I will get very sick, and I get very sad when I think of all the other children and babies who are sick with AIDS. I just wish that the government can start giving azidothymidine to pregnant HIV mothers to help stop the virus being passed on to their babies.

Nkosi Johnson aged 11 years, Durban, AIDS2000 (Nkosi succumbed to AIDS 6 years later).

It is hard to believe that, just 2 decades ago, in most of the world, 1 in 3 babies born to HIV-positive women was HIV-infected, and of those, 1 in 2 died by their second birthday leading to an estimated 5 million children dying of HIV since the beginning of the epidemic. The miracle of antiretroviral therapy (ART) has changed those dismal statistics, not only as direct treatment of adults, adolescents, and children living with HIV infection has kept them alive and from falling ill, but also as universal treatment of women living with HIV infection has prevented transmission of HIV to children prenatally, during birth, or during breastfeeding.

Since 1995, an estimated 1.6 million new HIV infections among children have been averted because of the provision of antiretroviral (ARV) medicines to women living with HIV during pregnancy and breastfeeding. Most of these infections (1.3 million) were averted between 2010 and 2015.^1^ Globally, the annual number of new infections among children (0–14 years) has almost halved since 2010 with a 47% reduction in new HIV cases.^2^

Despite this significant progress, the number of children becoming newly infected with HIV remains unacceptably high. In 2016, 24% [40%–12%] of pregnant women living with HIV did not have access to ARV medicines to prevent transmission to their infants.^3^ In the same year, around 160,000 [100,000–220,000] children became infected with HIV.^3^ In many of the most burdened countries, half of the children exposed to HIV were not tested within the recommended first 2 months of life, and half of the almost 2 million children living with HIV were still not receiving life-saving ART.^4^

As ART has reached increasing numbers of children, especially from an early age, mortality in perinatally HIV-infected children has declined dramatically, resulting in these children now surviving into adolescence and young adulthood. In addition, 610,000 adolescents (15–24 years) were infected with HIV in 2016, predominantly through sexual transmission, bringing the total number of adolescents living with HIV to 2.1 million.^2^ Rates of HIV counseling and testing, linkage, and adherence to care and retention in care all remain suboptimal in most regions among adolescent populations. Today, adolescents are the only population group for whom HIV-related mortality continues to increase.^5^

Early identification, prompt treatment, and effective monitoring and care for infants, children, and adolescents living with HIV can enable them to live long and fulfilling lives. However, a lack of necessary investment, resources and research to optimize testing, pediatric ARV medicines, and adolescent-friendly HIV services mean children and adolescents are in danger of being left behind.

For this reason, the undertaking by WHO and the Collaborative Initiative for Pediatric HIV Education and Research (CIPHER) to set a global prioritized research agenda for children and adolescents described in this journal series comes at an excellent time. The methods for this collaborative effort are described by Irvine et al,^6^ and the recommendations' research priorities for testing, treatment, and care among children and adolescents are described by Penazzato et al^7^ and Armstrong et al,^8^ respectively. The important additional ethical and legal requirements for conducting research among the most vulnerable can make such research more challenging and may contribute to children and adolescents living with HIV being left out of the research agenda. Oliveras and coauthors^9^ point out that this work is not only feasible but recommended if some very basic principles are followed. Getting the work done will need a good mix of innovation, opportunism, and careful design. Sohn and colleagues^10^ explain how using already existing data, much of it observational, can already teach us much about the best ways to care for these populations. As the number of new perinatal infections continues to decline, innovative designs will be required to test novel treatments and strategies—Ford and colleagues^11^ examine varied designs that have served to help answer challenging questions in the past and can help us to plan studies more strategically in the future. Clinical trials may not always be feasible or generalizable enough to address all the research questions raised in the collaborative initiative, and Ciaranello^12^ and Mark and colleagues^13^ explore the utility of modeling and implementation science, respectively, as additional ways to find answers to these questions.

We now have a research agenda laid out, and using the array of methods recommended above, we can fill the gaps in our knowledge that will bring us closer to ensuring that children and adolescents are not left behind. This will require strong political and financial commitment as well as an effective collaboration among key stakeholders, including academic institutions, national governments, community-based organizations, and interested donors. None of us will do this alone, and global platforms such as the one provided by Start Free—Stay Free—AIDS Free can catalyze the attention and the resources to make this happen for children and adolescents worldwide.^14^
